# Radiographic evaluation of segmental motion of scoliotic wedging segment in degenerative lumbar scoliosis

**DOI:** 10.1186/1748-7161-10-S1-P22

**Published:** 2015-01-19

**Authors:** Hiroyuki Yasuda, Akira Matsumura, Hidetomi Terai, Hiromitsu Toyoda, Akinobu Suzuki, Sho Dohzono, Kohji Tamai, Hiroaki Nakamura

**Affiliations:** 1 Osaka General Hospital of West Japan Railway Company, Japan; 2 Osaka City General Hospital, Japan; 3 Osaka City University Graduate School of Medicine, Japan

## Objective

Evaluation of segmental instability in DLS is essential for spinal surgery to make the decision "fusion or not fusion." Some previous studies have shown radiographic data regarding segmental instability in DLS. However, little is known about segmental motion of wedging segments in DLS. To evaluate the segmental instability of wedging segments using dynamic films and the impact of osteophyte formation on segmental motion in degenerative lumbar scoliosis (DLS).

## Methods

A total of 101 patients complaining of neurogenic claudication owing to lumbar spinal canal stenosis (LCS) were enrolled and divided into 2 groups based on their coronal spinal deformity; Cobb angle of >10 degree (DLS group) or <10 degree (LCS group). The following parameters were measured on anteroposterior films: lateral translation, degree of osteophyte formation, and range of motion (ROM) in lateral bending films. The facet joint space discrepancy was measured on computed tomography images. The radiographic parameters were compared between 85 wedging segments (>5 degrees) in DLS group and 150 nonwedging segments in LCS group.

## Results

Mean lateral translation was significantly greater in the DLS group (P<0.01 for each level). Average joint space discrepancy was significantly greater in the DLS group (P<0.01 at L2/3 and L3/4, P =0.01 at L4/5). ROM was significantly greater in the DLS group with the same degree osteophyte formation. Degree of osteophyte formation was inversely correlated with ROM in the DLS group (R(2)=0.5696).

## Conclusions

The present study indicated that wedging segments in DLS had greater motion than nonwedging segments in LCS. However, the osteophyte formation provided restabilization for the wedging segments in DLS, suggesting that evaluation of osteophyte formation is an important factor in surgical decision making for DLS.

